# Can MRI differentiate between atypical cartilaginous tumors and high-grade chondrosarcoma? A systematic review

**DOI:** 10.1080/17453674.2020.1763717

**Published:** 2020-05-20

**Authors:** Claudia Deckers, Maarten J Steyvers, Gerjon Hannink, H W Bart Schreuder, Jacky W J de Rooy, Ingrid C M Van Der Geest

**Affiliations:** a Department of Orthopedics, Radboud University Medical Center; Nijmegen;; b Department of Radiology, Radboud University Medical Center, Nijmegen;;; c Department of Operating Rooms, Radboud University Medical Center, Nijmegen, The Netherlands

## Abstract

Background and purpose — Adequate staging of chondroid tumors at diagnosis is important as it determines both treatment and outcome. This systematic review provides an overview of MRI criteria used to differentiate between atypical cartilaginous tumors (ACT) and high-grade chondrosarcoma (HGCS).

Patients and methods — For this systematic review PubMed and Embase were searched, from inception of the databases to July 12, 2018. All original articles describing MRI characteristics of pathologically proven primary central chondrosarcoma and ACT were included. A quality appraisal of the included papers was performed. Data on MRI characteristics and histological grade were extracted by 2 reviewers. Meta-analysis was performed if possible. The study is registered with PROSPERO, CRD42018067959.

Results — Our search identified 2,132 unique records, of which 14 studies were included. 239 ACT and 140 HGCS were identified. The quality assessment showed great variability in consensus criteria used for both pathologic and radiologic diagnosis. Due to substantial heterogeneity we refrained from pooling the results in a meta-analysis and reported non-statistical syntheses. Loss of entrapped fatty marrow, cortical breakthrough, and extraosseous soft tissue expansion appeared to be present more often in HGCS compared with ACT.

Interpretation — This systematic review provides an overview of MRI characteristics used to differentiate between ACT and HGCS. Future studies are needed to develop and assess more reliable imaging methods and/or features to differentiate ACT from HGCS.

The incidence of chondrosarcoma of bone appears to have been increasing during the last decade and is now reported to be the most common primary malignant bone tumor in several countries (Thorkildsen et al. 2018, van Praag et al. 2018). Conventional chondrosarcoma is the most common subtype of chondrosarcoma. Other subtypes of chondrosarcoma (e.g., juxtacortical, mesenchymal, or secondary chondrosarcoma) are rare and show different radiologic appearance and clinical behavior (Bindiganavile et al. 2015).

Conventional chondrosarcoma is classified into the histological grades 1 (currently known as atypical cartilaginous tumor [ACT]), 2, and 3. The metastatic potential, and therefore the disease-specific survival, correlates with the histological grade (Fletcher et al. 2013, Laitinen et al. 2018, Thorkildsen et al. 2018). ACTs rarely metastasize and are therefore reclassified as an intermediate type of tumor, not a malignancy (Fletcher et al. 2013). Due to the increase in patients undergoing MRI examinations for joint-related complaints, the incidental detection of ACT has increased substantially (van Praag et al. 2018).

With the increasing incidence of ACT, clear radiologic criteria to differentiate ACT from high-grade chondrosarcoma (i.e., grades 2 and 3) become more and more important. Adequate staging of chondroid tumors at diagnosis is important as it determines both treatment and prognosis. High-grade chondrosarcomas behave aggressively. Between 10% and 30% of grade 2 and about 70% of grade 3 chondrosarcomas metastasize (Evans et al. 1977). Hence, high-grade chondrosarcoma (HGCS) requires wide en bloc resection with free surgical margins. In contrast, ACTs are intermediate tumors and can be treated either with intralesional curettage and local adjuvant or nonoperatively with regular follow-up when located in the long bones (Deckers et al. 2016).

Due to the heterogenous composition of chondroid tumors, diagnostic biopsy is unreliable in assessing the genuine histological grade and malignant potential of chondrosarcomas (Laitinen et al. 2018). Therefore, physicians need to rely on imaging and clinical findings (e.g., pain is more common in HGCS) to differentiate ACT from HGCS. Imaging evaluation of cartilaginous and other bone tumors is generally based on multimodal assessment including at least conventional radiography and MRI (Nascimento et al. 2014).

During the most recent decades research has focused mainly on differentiating enchondroma from chondrosarcoma (Choi et al. 2013, Douis et al. 2014, Crim et al. 2015, Lisson et al. 2018). New insights have shown that both enchondroma and ACT located in the long bones can be observed without treatment (Deckers et al. 2016, Sampath Kumar et al. 2016, Chung et al. 2018). These insights make the differentiation between ACT and HGCS clinically relevant. Currently, literature on differentiating ACT from HGCS is sparse and clear radiologic criteria are lacking. Therefore, we performed a systematic review to provide an overview of MRI characteristics used to date to differentiate between ACT and HGCS.

## Methods

The aim of this systematic review is to provide an overview of MRI characteristics used to differentiate between atypical cartilaginous tumors (ACT) and high-grade chondrosarcoma (HGCS). The inclusion criteria and method of analysis were specified in advance and documented in a PROSPERO protocol (CRD42018067959). This study was conducted and reported according to PRISMA (Preferred Reporting Items for Systematic Reviews and Meta-Analyses) and MOOSE guidelines.

### Search strategy and selection of studies

The search strategy, composed of 3 elements (histology, MRI, and chondrosarcoma), was developed in collaboration with information specialists from the medical library of the Radboud University Medical Center Nijmegen, the Netherlands. The detailed search strategy can be found in Table 1 (see Supplementary data). No limits (e.g., language or publication date) were used.

**Table 3.** Study characteristics

Study	Study setting	Patients (n)	Tumor location (n)	MRI field strength	Intravenous contrast	MRI characteristics assessed
**Conventional MRI**						
Crim et al. 2015	Retrospective	12 CS 1	Humerus (5), radius (1), femur (4), fibula (2)	NR	+	Length, deep endosteal scalloping, cortical breakthrough, soft tissue mass, gadolinium enhancement
Douis et al. 2014	Retrospective	28 ACT 79 CS 1 36 CS 2 13 CS 3 23 Dediff a	Humerus (58), femur (98), tibia (24)	NR	–	Bone marrow edema, soft tissue edema, bone expansion, cortical thickening, cortical destruction, active periostitis, soft tissue mass, tumor length
Douis et al. 2018^b^	Retrospective 1980–2016	15 CS 1 3 CS 2 1 CS 3 4 Dediff a	Humerus (10), femur (9), tibia (3), fibula (1)	3T	+	Tumor length, endosteal scalloping, bone marrow edema, soft tissue edema, cortical destruction, periosteal reaction, bone expansion, macroscopic fat, calcification, soft tissue mass, hemorrhage
Errani et al. 2017	Retrospective 1986–2015	17 ACT	Humerus (5), femur (9), tibia (3)	1.5T	NR	Scalloping, soft tissue mass
Fayad et al. 2015	Retrospective 1991–2014	6 CS 2 1 CS 3	Hands and feet (7)	1.5T	+	T1 signal ^c^, T1 heterogeneity ^c^, T2 hyperintense ^c^, T2 heterogeneity ^c^, bone marrow edema, soft tissue edema, gadolinium enhancement, soft tissue mass
Kang et al. 2016	Retrospective 1993–2016	6 CS 1 15 HGCS	Para-acetabular (21)	1.5T	+	Length, high signal foci on T1 ^c^, high signal on T1–T2-STIR ^c^, soft-tissue mass, peritumoral edema, lobular border, acetabular cartilage destruction ^c^, diffuse signal changes in acetabulum ^c^, mass inside hip joint ^c^, femoral head involvement ^c^
Liu et al. 2017	Retrospective 2008–2015	17 Dediff a	NR	3T	+	Patterns of bone destruction, periosteal reaction, matrix mineralization, soft tissue mass, enhancement pattern, signal intensity
MacSweeney et al. 2003	Retrospective 1995–2005	8 Dediff a	Humerus (2), femur (6)	1.0 or 1.5T	+	Soft tissue extension
Yoo et al. 2009	Retrospective 1999–2008	28 LG 14 HG	Humerus (16), scapula (1), pelvic bone (9), femur (15), fibula (1)	1.0T or 1.5T	+	T1 signal ^c^, entrapped fat within the tumor, lobular architecture preservation, cortical destruction, soft tissue mass, gadolinium enhancement
Yoshimura et al. 2013	Retrospective 1996–2011	6 CS 1 10 CS 2 1 CS 3 rib (1)	Humerus (4), ulna (1), phalange (2), femur (7), tibia (1), calcaneus (1),	NR	+	Entrapped fat within the tumor, lobular architecture, ring and arc enhancement, T1 signal ^c^, soft tissue mass, gadolinium enhancement
**Diffusion weighted imaging**						
Douis et al. 2015	Retrospective 2012–2013	5 ACT 15 CS 1 3 CS 2 2 CS 3 3 Dediff a	Humerus (19), rib (2), hand (3), spine (1), pelvis (5), femur (17), tibia (5) ^d^	3T	–	Apparent diffusion coefficient
Müller et al. 2016	Retrospective 2007–2012	8 CS 1	Skull base	NR	NR	Apparent diffusion coefficient
Welzel et al. 2018	Retrospective 2009–2014	24 CS 1 10 CS 2 1 CS 3	Skull base	3T	+	Apparent diffusion coefficient
**Dynamic contrast-enhanced MRI**						
Douis et al. 2018	Retrospective 1980–2016	15 CS 1 3 CS 2 1 CS 3 4 Dediff a	Humerus (10), femur (9), tibia (3), fibula (1)	3T	+	Dynamic contrast-enhanced (DCE) MRI parameters; angle of DCE-MRI curve, absolute enhancement and relative enhancement
**Quantitative texture analysis**						
Lisson et al. 2018	Retrospective	11 CS1	NR	1.5 & 3T	+	Quantitative texture analysis to assess tumor heterogeneity

NR = not reported.

**^a^** Dedifferentiated chondrosarcoma.

**^b^** Study mentioned twice as different imaging modalities are used in the same study.

**^c^** MRI characteristic not analyzed in our systematic review.

**^d^** 24 enchondroma tumors are included in description of tumor location.

The search strategy was carried out in Pubmed and Embase (last search performed July 12, 2018). Additionally, reference lists of the included studies and of relevant reviews were screened for potentially relevant papers.

After removal of duplicates, all unique records were imported into EROS (Early Review Organizing Software, Buenos Aires, Argentina) to allocate references randomly to 2 independent reviewers (CD, MS) responsible for screening and selection. Discrepancies were solved by discussion.

During the first screening phase, original studies (i.e., no case reports, conference proceedings, systematic reviews) were included if they mentioned the combination of chondrosarcoma, histology/pathology, and imaging in title and/or abstract. If not enough information was provided to make a valid judgment, the full text was evaluated. Full-text versions of all selected studies were screened and included if they met the pre-specified eligibility criteria: (1) preoperative MRI grading; (2) histopathological grading; (3) presence of MRI characteristics per chondrosarcoma grade; (4) primary central chondrosarcoma of bone; (5) adult patients.

Types other than primary central chondrosarcoma of bone (e.g., juxtacortical, mesenchymal, or secondary chondrosarcoma) were excluded as these different types of tumor show different radiologic appearance and clinical behavior (Bindiganavile et al. 2015).

### Data extraction

2 independent reviewers (CD, MS) performed data extraction from each included study in a pre-piloted form. Information was extracted related to: study design, studied population, tumor location and size, tumor grade based on postoperative histology/pathology, pathology criteria used for diagnosis, type of MRI used, and MRI characteristics described per grade of chondrosarcoma (e.g., cortical breakthrough, soft tissue expansion).

If studies included other types of chondrosarcoma (e.g., juxtacortical, mesenchymal, or secondary chondrosarcoma), only data related to central ACT and high-grade chondrosarcoma were extracted.

If outcome data were presented incompletely, we tried to contact the authors to obtain the original data. A reminder was sent to those who did not reply within 2 weeks. When attempts to obtain original data failed, the article was excluded.

According to the WHO classification, ACTs (i.e., chondrosarcoma grade 1) were categorized as low-grade chondrosarcoma (LGCS). Grade 2, grade 3 and dedifferentiated chondrosarcomas were categorized as HGCS (Fletcher et al. 2013).

### Quality appraisal

The quality of the included studies was assessed using STROBE for the assessment of observational studies (Table 2, see Supplementary data). We are aware of the fact that the authors of STROBE did not develop their tool for methodological quality assessment. However, due to the lack of validated and accepted tools for such assessments of observational studies, STROBE is often used for this purpose (da Costa et al. 2011). In accordance with other studies, only 10 of the 22 items of the STROBE checklist were used for methodological assessment (da Costa et al. 2011, Shemesh et al. 2017). The other 12 of the 22 items were found not to contribute to the methodological assessment.

In addition, we analyzed the quality of histopathology and MRI assessments. We checked whether there was (1) a description of the criteria used for diagnosis, (2) cited reference to consensus criteria used for diagnosis, and (3) if the diagnosis was established by an experienced musculoskeletal pathologist/radiologist (Shemesh et al. 2017). In addition, we added whether the pathologist and/or radiologist was blinded. If the level of experience of the pathologist/radiologist was not specified in the article, the authors were contacted.

2 reviewers (CD, MS) independently scored each item as: well described (+), partly described (±), or poorly/not described (–). Discrepancies were solved by discussion.

No overall score was calculated, as we felt different study characteristics that are related to study quality cannot be judged as if they are of equal importance or interchangeable (Ioannidis 2011).

### Data analysis

Heterogeneity was assessed by visual inspection of forest plots and quantified using the I^2^ and τ^2^. The latter were calculated even when the judgment was made that calculating a pooled estimate was not justifiable (Higgins et al. 2003). Before undertaking a meta-analysis, we first checked whether the studies were similar enough to justify combining their results. If the features of studies were deemed not sufficiently similar to combine in a meta-analysis, we displayed the results of included studies in a forest plot but suppressed the summary estimate (Faber et al. 2016, Mueller et al. 2018, Reeves et al. 2019). If possible, pooled estimates of proportions with their corresponding 95% confidence intervals were calculated using the logit transformation using inverse-variance weighting within a random effects model framework. Between-study variance was quantified using the τ^2^ statistic, estimated using the Sidik-Jonkman estimator. Data were analyzed using R version 3.4.3 (R Foundation for Statistical Computing, Vienna, Austria) using the meta package.

Publication bias was assessed only if more than 10 studies were included in the meta-analysis.

Data for different MR modalities (conventional MRI, diffusion-weighted imaging, dynamic contrast enhancement, and quantitative texture analysis) were reported separately, as these outcome measures were found not to be comparable to pool.

MRI signal intensity, such as high signal on T1, can be related to several histopathological findings (e.g., hemorrhage, entrapped fat) and therefore does not necessarily indicate grade of chondrosarcoma. Therefore, we have chosen to exclude these MRI characteristics from our analysis.

### Funding and potential conflicts of interest

There was no funding source for this study. None of the authors reported any conflict of interest.  

## Results

**Figure 1.**
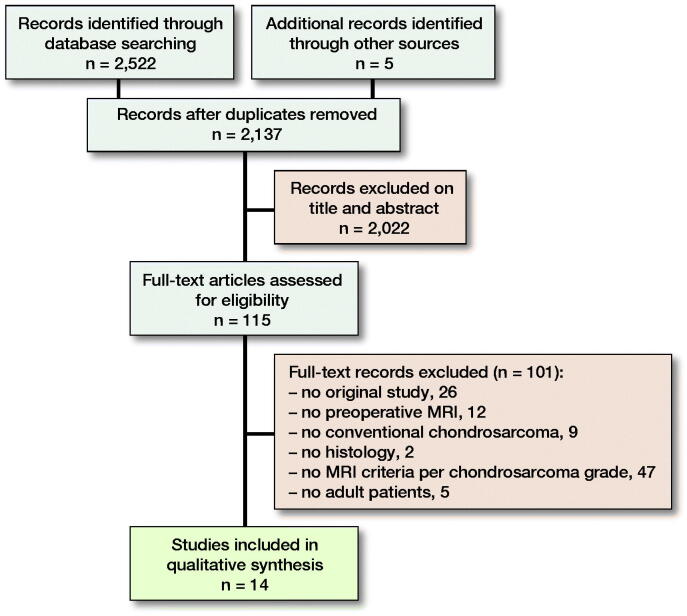
PRISMA flow diagram.

**Figure 2.**
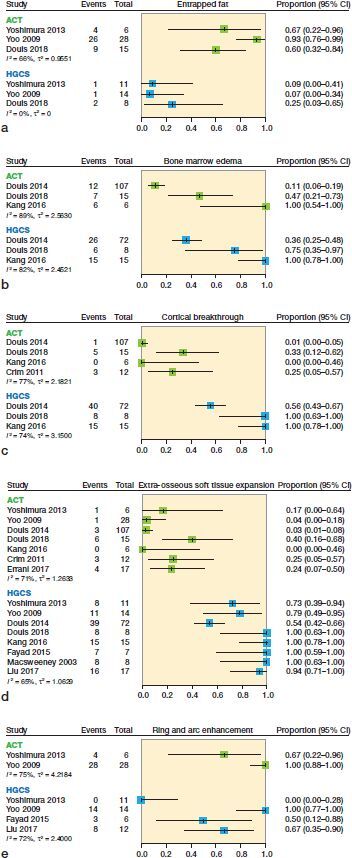
Forest plots of proportions of the reported presence of (a) entrapped fat, (b) bone marrow edema, (c) cortical breakthrough, (d) extra-osseous soft tissue expansion, and (e) ring and arc enhancement on conventional MRI in atypical cartilaginous tumors (ACT) and high-grade chondrosarcoma (HGCS).

Conducting our search strategy in PubMed and Embase retrieved 2,132 unique records. 5 additional relevant articles were found via cross-referencing. 2,123 articles were excluded because they did not meet our eligibility criteria (Figure 1). Errani et al. (2017) provided additional data on request. Consequently 14 articles were included in our systematic review (Table 3). 239 ACT and 140 HGCS were included in this systematic review. The following conventional MRI characteristics were reported by the included studies and analyzed: entrapped fat, perilesional bone marrow edema, internal lobular architecture, lobular outer margin, bone expansion, cortical thickening, scalloping, cortical breakthrough, periosteal edema, soft tissue edema, extra-osseous soft tissue expansion, ring and arc enhancement, solid enhancement, and central non-enhancing region. Due to substantial heterogeneity (I^2^ 50–90%) and insufficient information to further investigate this heterogeneity we decided to refrain from pooling the results and only provide non-statistical syntheses. The reported presence of conventional MRI characteristics in both ACT and HGCS is displayed in separate forest plots but we suppressed the summary estimates (Figure 2). Only the most commonly reported MRI characteristics are shown in Figure 2; all other MRI characteristics can be found in Figure 3 (see Supplementary data).

Both Kang et al. (2016) and Douis et al. (2018) compared maximum tumor size between ACT and HGCS. Kang et al. found a significant difference in tumor length between ACT (3.0 cm, SD 0.7 cm) and HGCS (7.4 cm, SD 2.7 cm), whereas Douis et al. did not find a difference in tumor length between ACT (11 cm, range 2.1–26 cm) and HGCS (13 cm, range 4.3–30 cm).

3 DWI studies were included describing apparent diffusion coefficient (ADC). Douis et al. (2015) found no statistically significant difference in both mean apparent diffusion coefficient (ADC) and minimum ADC between ACT and high-grade chondrosarcoma.

Welzel et al. (2018) found in their subgroup analyses that chondrosarcoma grade 1 had statistically significantly higher, mean, minimum, maximum, and normalized ADC values than grade 2 chondrosarcoma in the skull base.

Müller et al. (2016) measured the following ADC values in 8 chondrosarcoma grade 1 tumors of the skull base: mean ADC 2017 (SD 140) × 10^−6^ mm^2^/s. No ADC values of high-grade chondrosarcoma were measured.

Only 1 study was found that described dynamic contrast-enhanced (DCE) MRI parameters.

Douis et al. (2018) found no statistically significant difference for the various DCE-MRI parameters (angle of the DCE-MRI curve, absolute enhancement, and relative enhancement on DCE MRI) between LGCS and HGCS.

Lisson et al. (2018) performed an MRI-based 3D texture analysis in which they compared enchondroma with low-grade chondrosarcoma. No comparison with high-grade chondrosarcoma was made. The most promising texture parameters for differentiation were, among others, kurtosis (the magnitude of pixel distribution) in the contrast-enhanced T1-weighted images and entropy in non-contrast T1-weighted images.

The quality appraisal of diagnosis is presented in Table 4 (see Supplementary data). The individual scored items on the STROBE checklist of each study can be found in the Supplementary data. Our assessment of the reporting quality shows great variability in consensus criteria used for diagnosis for both pathologic and radiologic diagnosis. Only in 7 of 14 studies did an experienced pathologist in musculoskeletal oncology perform pathologic assessment. In the other 7 studies level of expertise was not mentioned. In 10 of 14 studies MRI assessment was performed by experienced musculoskeletal radiologists.  

## Discussion

Correct diagnosis of chondrosarcoma grade is crucial in determining both treatment and prognosis. Therefore, we performed a systematic review to provide an overview of MRI characteristics used to differentiate between ACT and high-grade chondrosarcoma.

Although we did not pool the overall results due to the considerable amount of heterogeneity, it appears that, compared with ACT, high-grade chondrosarcoma may present more often with the following MRI characteristics: loss of entrapped fatty marrow, cortical breakthrough, and extraosseous soft tissue expansion.

These MRI findings are in line with the histopathological findings described by several authors (Brien et al. 1997, Yoo et al. 2009, Logie et al. 2013).

In cartilaginous tumors production of chondroid matrix results in the typical lobulated growth pattern and the so-called ring and arc appearance (Logie et al. 2013). In HGCS these typical chondroid features become lost due to poor differentiation of cells. Chondrosarcoma cells actively infiltrate between individual fat cells, compressing and eventually replacing them (Brien et al. 1997). Absence of areas of entrapped fat is therefore highly indicative of HGCS. In addition, invasion of Haversian systems leads to periosteal reaction. Eventually there is destruction of the cortex and invasion of soft tissue (Brien et al. 1997). Yoo et al. (2009) found that on gross pathological evaluation, a central non-enhancing region corresponded to an area of hemorrhagic cyst, necrosis, and/or yellow-brown soft tissue mass reflecting a myxoid change, all characteristics of malignant tumors.

Due to the heterogeneity of cartilage tumors, areas of ACT can be seen in HGCS lesions. Therefore, the presence of MRI characteristics indicating ACT must be viewed in context and clinical findings must be taken into account. In addition, single MRI characteristics alone cannot differentiate between ACT and HGCS.

The assessment of the clinical relevance of our findings is not straightforward. Heterogeneity was substantial (I2 50–90%) in the majority of the analyses. Due to the considerable heterogeneity we decided not to perform a meta-analysis. Heterogeneity may be explained by either clinical and/or methodological diversity between included studies. Included studies showed great variability in tumor location within and between studies. Different bones (e.g., phalanges, femur) as well as types of bone (e.g., flat, long bones) were included in most studies, which might show different clinical behavior and radiologic appearance (Bindiganavile et al. 2015). We were unable to perform a sensitivity analysis on tumor location. In addition, heterogeneity might be caused by poor reliability between radiologists. The SLICED study group showed poor to slight reliability between radiologists for the subgroup of outcome-determined high-risk patients (SLICED Study Group 2007). However, the imaging modalities available for radiologists varied and different criteria were used. In those cases where MRI scans were available the reliability increased substantially. Zamora et al. (2017) showed fair interobserver agreement between orthopedic oncologists for diagnosis and grading of cartilaginous neoplasms. Nevertheless, no evaluator proposed observation or follow-up for lesions considered to be a malignant neoplasm.

### Limitations

To reduce bias we excluded tumors other than primary central chondrosarcoma from our systematic review. Several studies were excluded as they included, e.g., secondary or periosteal chondrosarcoma as well and we were not able to extract data on the primary central chondrosarcoma (Varma et al. 1992, Geirnaerdt et al. 1993, De Beuckeleer et al. 1995, Geirnaerdt et al. 2000, Fritz et al. 2018). Excluding studies to reduce bias resulted in a limited number of tumors being included in this systematic review.

Several studies have shown that both radiological and histopathological diagnosis of chondrosarcoma is subject to low reproducibility, which may be caused by difficult and ambiguous definitions (SLICED Study Group 2007, Zamora et al. 2017). Different terminology has been used in chondrosarcoma literature during the past years, for example CLUMP (cartilaginous lesion of unknown malignant potential), borderline chondrosarcoma, or grade 0.5 CS, compromising comparability of studies. As can be seen in Table 4, several different grading methods have been used to assess the level of malignancy of chondrosarcoma. In addition, other imaging methods used (e.g., radiographs, CT) could have influenced the radiologist during MRI interpretation. Only Crim et al. (2015) and Fayad et al. (2015) stated that both radiographs and MRI were available for the radiologist. Other articles included did not report information on other imaging methods used but this could have been the case as combining different imaging methods is common practice.

Possible interreader variability of chondrosarcoma grading may have resulted in misclassification bias in our systematic review. We would recommend a standardized grading method and terminology for chondroid tumors to improve comparability between studies and decrease the amount of bias.

Third, we are aware of the fact that the authors of STROBE did not develop their tool for methodological quality assessment. Due to the lack of validated and accepted tools for such assessments for observational studies, STROBE is often used for this purpose (da Costa et al. 2011). We have used relevant items of the STROBE tool to give an overview of the methodology through the included papers. As shown by Mueller et al. (2018) there is considerable disagreement on how systematic reviews of observational studies should be done. We agree that there is a need for a comprehensive source of methodological guidance, in particular for quality assessment of observational studies.

This systematic review provides an overview of currently used MRI characteristics. Future studies are needed to develop and assess a reliable method for differentiating chondrosarcoma based on radiologic and clinical findings. Reliability could be increased by protocol-driven image acquisition for cartilaginous lesions and an easy to use grading system that could be reliably quantified.

From this systematic review it appears that MRI may possibly be helpful to differentiate ACT from HGCS. Extraosseous soft tissue expansion and cortical breakthrough appear to be present more often in HGCS and entrapped fat presents more often in ACT.

As a correct differentiation of ACT and HGCS is important, we recommend future studies to develop and assess more reliable imaging methods and/or features to differentiate ACT from HGCS.

## Supplementary Material

Supplemental Material

## Supplementary data

Tables 1, 2, and 4 and Figure 3 are available as supplementary data in the online version of this article, http://dx.doi.org/10.1080/17453674.2020.1763717

## References

[CIT0001] Aoki J, Sone S, Fujioka F, Terayama K, Ishii K, Karakida O, Imai S, Sakai F, Imai Y. MR of enchondroma and chondrosarcoma: rings and arcs of Gd-DTPA enhancement. J Comput Assist Tomogr 1991; 15(6): 1011–6. 1939751 10.1097/00004728-199111000-00021

[CIT0002] Bindiganavile S, Han I, Yun J Y, Kim H-S. Long-term outcome of chondrosarcoma: a single institutional experience. Cancer Res Treat 2015; 47(4): 897. 25687868 10.4143/crt.2014.135 PMC4614192

[CIT0003] Brien E W, Mirra J M, Kerr R. Benign and malignant cartilage tumors of bone and joint: their anatomic and theoretical basis with an emphasis on radiology, pathology and clinical biology. Skeletal Radiol 1997; 26(6): 325–53. 9229417 10.1007/s002560050246

[CIT0004] Choi B-B, Jee W-H, Sunwoo H-J, Cho J-H, Kim J-Y, Chun K-A, Hong S-J, Chung H W, Sung M-S, Lee Y-S. MR differentiation of low-grade chondrosarcoma from enchondroma. Clin Imaging 2013; 37(3): 542–7. 23041161 10.1016/j.clinimag.2012.08.006

[CIT0005] Chung B M, Hong S H, Yoo H J, Choi J-Y, Chae H-D, Kim D H. Magnetic resonance imaging follow-up of chondroid tumors: regression vs. progression. Skeletal Radiol 2018; 47(6): 755–61. 29197957 10.1007/s00256-017-2834-z

[CIT0006] Crim J, Schmidt R, Layfield L, Hanrahan C, Manaster B J. Can imaging criteria distinguish enchondroma from grade 1 chondrosarcoma? Eur J Radiol 2015; 84(11): 2222–30. 26220916 10.1016/j.ejrad.2015.06.033

[CIT0007] da Costa B R, Cevallos M, Altman D G, Rutjes A W, Egger M. Uses and misuses of the STROBE statement: bibliographic study. BMJ open 2011:2010–000048. 10.1136/bmjopen-2010-000048 PMC3191404 22021739

[CIT0008] Dahlin D C, Henderson E D. Chondrosarcoma, a surgical and pathological problem: review of 212 cases. J Bone Joint Surg 1956; 38(5): 1025–125. 13367080

[CIT0009] Dahlin D C, Beabout J W. Dedifferentiation of low-grade chondrosarcomas. Cancer 1971; 28(2): 461–6. 5566365 10.1002/1097-0142(197108)28:2<461::aid-cncr2820280227>3.0.co;2-u

[CIT0010] De Beuckeleer L H, De Schepper A M, Ramon F, Somville J. Magnetic resonance imaging of cartilaginous tumors: a retrospective study of 79 patients. Eur J Radiol 1995; 21(1): 34–40. 8654456 10.1016/0720-048x(96)81067-9

[CIT0011] De Beuckeleer L H, De Schepper A, Ramon F. Magnetic resonance imaging of cartilaginous tumors: is it useful or necessary? Skeletal Radiol 1996; 25(2): 137–41. 8848742 10.1007/s002560050050

[CIT0012] Deckers C, Schreuder B H, Hannink G, de Rooy J W, van der Geest I C. Radiologic follow-up of untreated enchondroma and atypical cartilaginous tumors in the long bones. J Surg Oncol 2016; 114(8): 987–91. 27696436 10.1002/jso.24465 PMC6222252

[CIT0013] Dorfman H D, Czerniak B, Bone tumors, in: H.D. Dorfman, B. Czerniak (Eds.), Benign Cartilage Lesions, Mosby, St. Louis, 1998, p. 263.

[CIT0014] Douis H, Singh L, Saifuddin A. MRI differentiation of low-grade from high-grade appendicular chondrosarcoma. Eur Radiol 2014; 24(1): 232–40. 24337911 10.1007/s00330-013-3003-y

[CIT0015] Douis H, Jeys L, Grimer R, Vaiyapuri S, Davies A. Is there a role for diffusion-weighted MRI (DWI) in the diagnosis of central cartilage tumors? Skeletal Radiol 2015; 44(7): 963–9. 25744812 10.1007/s00256-015-2123-7

[CIT0016] Douis H, Parry M, Vaiyapuri S, Davies A. What are the differentiating clinical and MRI-features of enchondromas from low-grade chondrosarcomas? Eur Radiol 2018; 28(1): 398–409. 28695356 10.1007/s00330-017-4947-0

[CIT0017] Enneking W F. A system of staging musculoskeletal neoplasms. Clin Orthop Rel Res 1986; (204):9–24. 3456859

[CIT0018] Errani C, Tsukamoto S, Ciani G, Akahane M, Cevolani L, Tanzi P, Kido A, Honoki K, Tanaka Y, Donati D M. Risk factors for local recurrence from atypical cartilaginous tumour and enchondroma of the long bones. Eur J Orthop Surg Traumatol 2017; 27(6): 805–11. 28501961 10.1007/s00590-017-1970-4

[CIT0019] Evans H L, Ayala A G, Romsdahl M M. Prognostic factors in chondrosarcoma of bone: a clinicopathologic analysis with emphasis on histologic grading. Cancer 1977; 40(2): 818–31. 890662 10.1002/1097-0142(197708)40:2<818::aid-cncr2820400234>3.0.co;2-b

[CIT0020] Faber T, Ravaud P, Riveros C, Perrodeau E, Dechartres A. Meta-analyses including non-randomized studies of therapeutic interventions: a methodological review. BMC Med Res Methodol 2016; 16(1): 35. 27004721 10.1186/s12874-016-0136-0 PMC4804609

[CIT0021] Fayad L M, Ahlawat S, Khan M S, McCarthy E. Chondrosarcomas of the hands and feet: a case series and systematic review of the literature. Eur J Radiol 2015; 84(10): 2004–12. 26189572 10.1016/j.ejrad.2015.06.026

[CIT0022] Fletcher C D M, Bridge J A, Hogendoorn P, Mertens F. WHO classification of tumours of soft tissue and bone. Lyon, France: IARC Press; 2013.

[CIT0023] Fritz B, Müller D A, Sutter R, Wurnig M C, Wagner M W, Pfirrmann C W, Fischer M A. Magnetic Resonance Imaging–Based Grading of Cartilaginous Bone Tumors: Added Value of Quantitative Texture Analysis. Invest Radiol 2018; 53(11): 663–72. 29863601 10.1097/RLI.0000000000000486

[CIT0024] Geirnaerdt M, Bloem J, Eulderink F, Hogendoorn P, Taminiau A. Cartilaginous tumors: correlation of gadolinium-enhanced MR imaging and histopathologic findings. Radiology 1993; 186(3): 813–7. 8430192 10.1148/radiology.186.3.8430192

[CIT0025] Geirnaerdt M, Hermans J, Bloem J L, Kroon H M, Pope T, Taminiau A, Hogendoorn P. Usefulness of radiography in differentiating enchondroma from central grade 1 chondrosarcoma. AJR Am J Roentgenol 1997; 169(4): 1097–104. 9308471 10.2214/ajr.169.4.9308471

[CIT0026] Geirnaerdt M J, Hogendoorn P C, Bloem J L, Taminiau A H, van der Woude H-J. Cartilaginous tumors: fast contrast-enhanced MR imaging. Radiology 2000; 214(2): 539–46. 10671608 10.1148/radiology.214.2.r00fe12539

[CIT0027] Henderson E D, Dahlin D C. Chondrosarcoma of bone—a study of two hundred and eighty-eight cases. J Bone Joint Surg 1963; 45(7): 1450–8. 14069783

[CIT0028] Higgins J P, Thompson S G, Deeks J J, Altman D G. Measuring inconsistency in meta-analyses. BMJ 2003; 327(7414): 557–60. 12958120 10.1136/bmj.327.7414.557 PMC192859

[CIT0029] Hogendoorn P C W, Bovee J, Nielsen G P. World health organizationclassification of tumours of soft tissue and bone. In: Fletcher C D M, Bridge J A, Hogendoorn P C W, Mertens F (Eds). IARCPress, Lyon 2013, pp 264–8.

[CIT0030] Inwards C Y, Unni K K. Classification and grading of bone sarcomas. Hematol Oncol Clin North Am 1995; 9(3): 545–70. 7649942

[CIT0031] Inwards C H P. In: Fletcher C D M, Bridge J A, Hogendoorn P C W, Mertens F (Eds.) World Health Organization Classification of Tumours of Soft Tissue and Bone. IARC Press, Lyon 2013, pp 269–270.

[CIT0032] Ioannidis J P. Commentary: adjusting for bias: a user’s guide to performing plastic surgery on meta-analyses of observational studies. Int J Epidemiol 2011; 40(3): 777–9. 21233141 10.1093/ije/dyq265

[CIT0033] Kang Y, Yuan W, Ding X, Wang G. Chondrosarcoma of the para-acetabulum: correlation of imaging features with histopathological grade. Radiol Med 2016; 121(12): 897–904. 27553036 10.1007/s11547-016-0673-y

[CIT0034] Laitinen M, Stevenson J, Parry M, Sumathi V, Grimer R, Jeys L. The role of grade in local recurrence and the disease-specific survival in chondrosarcomas. Bone Joint J 2018; 100(5): 662–6. 29701096 10.1302/0301-620X.100B5.BJJ-2017-1243.R1

[CIT0035] Lisson C S, Lisson C G, Flosdorf K, Mayer-Steinacker R, Schultheiss M, von Baer A, Barth T F, Beer A J, Baumhauer M, Meier R. Diagnostic value of MRI-based 3D texture analysis for tissue characterisation and discrimination of low-grade chondrosarcoma from enchondroma: a pilot study. Eur Radiol 2018; 28(2): 468–77. 28884356 10.1007/s00330-017-5014-6

[CIT0036] Liu C, Xi Y, Li M, Jiao Q, Zhang H, Yang Q, Yao W. Dedifferentiated chondrosarcoma: radiological features, prognostic factors and survival statistics in 23 patients. PloS One 2017; 12(3): e0173665. 28301537 10.1371/journal.pone.0173665 PMC5354284

[CIT0037] Logie C I, Walker E A, Forsberg J A, Potter B K, Murphey M D. Chondrosarcoma: a diagnostic imager’s guide to decision making and patient management. In: Seminars in Musculoskeletal Radiology. New York: Thieme Medical Publishers; 2013. p 101–15. 10.1055/s-0033-1342967 23673542

[CIT0038] MacSweeney F, Darby A, Saifuddin A. Dedifferentiated chondrosarcoma of the appendicular skeleton: MRI-pathological correlation. Skeletal Radio 2003; 32(12): 671–8. 10.1007/s00256-003-0706-1 14586574

[CIT0039] Mercuri M, Campanacci L. Dedifferentiated chondrosarcoma. Skeletal Radiol 1995; 24(6): 409–16. 7481896 10.1007/BF00941235

[CIT0040] Mirra M, Gold R, Downs J, Eckardt J J. A new histologic approach to the differentiation of enchondroma and chondrosarcoma of the bones. A clinicopathologic analysis of 51 cases. Clin Otyhop rel Res 1985; (201): 214–37. 4064409

[CIT0041] Mirra J M, Picci P, Gold R H. Bone Tumors: Clinical, Radiologic, andPathologic Correlations, Lea & Febiger, Philadelphia, 1989.

[CIT0042] Mueller M, D’Addario M, Egger M, Cevallos M, Dekkers O, Mugglin C, Scott P. Methods to systematically review and meta-analyse observational studies: a systematic scoping review of recommendations. BMC Med Res Methodol 2018; 18(1): 44. 29783954 10.1186/s12874-018-0495-9 PMC5963098

[CIT0043] Müller U, Kubik-Huch R A, Ares C, Hug E B, Löw R, Valavanis A, Ahlhelm F J. Is there a role for conventional MRI and MR diffusion-weighted imaging for distinction of skull base chordoma and chondrosarcoma? Acta Radiol 2016; 57(2): 225–32. 25722460 10.1177/0284185115574156

[CIT0044] Murphey M D, Flemming D J, Boyea S R, Bojescul J A, Sweet DE, Temple H T. Enchondroma versus chondrosarcoma in the appendicular skeleton: differentiating features. Radiographics 1998; 18(5): 1213–37. 9747616 10.1148/radiographics.18.5.9747616

[CIT0045] Murphey M D, Walker E A, Wilson A J, Kransdorf M J, Temple H T, Gannon F H. From the archives of the AFIP: imaging of primary chondrosarcoma: radiologic-pathologic correlation. Radiographics 2003; 23(5): 1245–78. 12975513 10.1148/rg.235035134

[CIT0046] Nascimento D, Suchard G, Hatem M, de Abreu A. The role of magnetic resonance imaging in the evaluation of bone tumours and tumour-like lesions. Insights Imaging 2014; 5(4): 419–40. 25005774 10.1007/s13244-014-0339-z PMC4141345

[CIT0047] Pritchard D J, Lunke R J, Taylor W F, Dahlin D C, Medley B E. Chondrosarcoma: a clinicopathologic and statistical analysis. Cancer 1980; 45(1): 149–57. 7350999 10.1002/1097-0142(19800101)45:1<149::aid-cncr2820450125>3.0.co;2-a

[CIT0048] Reeves B C, Deeks J J, Higgins J P, Shea B, Tugwell P, Wells G A, Group CNRSoIM. Including non-randomized studies on intervention effects. Cochrane Handbook for Systematic Reviews of Interventions 2019: 595–620.

[CIT0049] Rozeman L, Cleton-Jansen A, Hogendoorn P. Pathology of primary malignant bone and cartilage tumours. Int Orthop 2006; 30(6): 437–44. 16944143 10.1007/s00264-006-0212-x PMC3172744

[CIT0050] Sampath Kumar V, Tyrrell P, Singh J, Gregory J, Cribb G, Cool P. Surveillance of intramedullary cartilage tumours in long bones. Bone Joint J 2016; 98(11): 1542–7. 27803232 10.1302/0301-620X.98B11.37864

[CIT0051] Sanerkin N. The diagnosis and grading of chondrosarcoma of bone a combined cytologic and histologic approach. Cancer 1980; 45(3): 582–94. 6928110 10.1002/1097-0142(19800201)45:3<582::aid-cncr2820450326>3.0.co;2-#

[CIT0052] Shemesh S, Acevedo-Nieves J, Pretell-Mazzini J. Treatment strategies for central low-grade chondrosarcoma of long bones: a systematic review of the literature and meta-analysis. Musculoskelet Surg 2017: 1–15. 10.1007/s12306-017-0507-7 28986742

[CIT0053] SLICED Study Group. Reliability of histopathologic and radiologic grading of cartilaginous neoplasms in long bones. J Bone Joint Surg Am 2007; 89(10): 2113–23. 17908885 10.2106/JBJS.F.01530

[CIT0054] Thorkildsen J, Taksdal I, Bjerkehagen B, Haugland H K, Børge Johannesen T, Viset T, Norum O-J, Bruland Ø, Zaikova O. Chondrosarcoma in Norway 1990–2013: an epidemiological and prognostic observational study of a complete national cohort. Acta Oncol 2018: 1–10. 30632866 10.1080/0284186X.2018.1554260

[CIT0055] van Praag V, Rueten-Budde A, Ho V, Dijkstra P, van der Geest I C, Bramer J A, Schaap G R, Jutte P C, Schreuder H B, Ploegmakers J. Incidence, outcomes and prognostic factors during 25 years of treatment of chondrosarcomas. Surg Oncol 2018; 27(3): 402–8. 30217294 10.1016/j.suronc.2018.05.009

[CIT0056] Varma D, Ayala A, Carrasco C, Guo S, Kumar R, Edeiken J. Chondrosarcoma: MR imaging with pathologic correlation. Radiographics 1992; 12(4): 687–704. 1636034 10.1148/radiographics.12.4.1636034

[CIT0057] Walden M J, Murphey M D, Vidal J A. Incidental enchondromas of the knee. AJR Am J Roentgenol 2008; 190(6):1 611-5. 10.2214/AJR.07.2796 18492914

[CIT0058] Welkerling H, Werner M, Delling G. Histologic grading of chondrosarcoma. A qualitative and quantitative analysis of 74 cases of the Hamburg bone tumor register. Pathologe 1996; 17(1): 18–25. 8685092 10.1007/s002920050130

[CIT0059] Welkerling H, Kratz S, Ewerbeck V, Delling G. A reproducible and simple grading system for classical chondrosarcomas. Analysis of 35 chondrosarcomas and 16 enchondromas with emphasis on recurrence rate and radiological and clinical data. Virchows Arch 2003; 443(6): 725–33. 14513378 10.1007/s00428-003-0896-x

[CIT0060] Welzel T, Meyerhof E, Uhl M, Huang K, von Deimling A, Herfarth K, Debus J. Diagnostic accuracy of DW MR imaging in the differentiation of chordomas and chondrosarcomas of the skull base: a 3.0-T MRI study of 105 cases. Eur J Radiol 2018; 105: 119–24. 30017267 10.1016/j.ejrad.2018.05.026

[CIT0061] Yoo H J, Hong S H, Choi J-Y, Moon K C, Kim H-S, Choi J-A, Kang H S. Differentiating high-grade from low-grade chondrosarcoma with MR imaging. Eur Radiol 2009; 19(12): 3008–14. 19547983 10.1007/s00330-009-1493-4

[CIT0062] Yoshimura Y, Isobe K-i, Arai H, Aoki K, Kito M, Kato H. Preoperative radiographic and histopathologic evaluation of central chondrosarcoma. Arch Orthop Trauma Surg 2013; 133(9): 1225–31. 23820853 10.1007/s00402-013-1800-z PMC3751216

[CIT0063] Zamora T, Urrutia J, Schweitzer D, Amenabar P P, Botello E. Do orthopaedic oncologists agree on the diagnosis and treatment of cartilage tumors of the appendicular skeleton? Clin Orthop Relat Res 2017; 475(9): 2176–86. 28205076 10.1007/s11999-017-5276-y PMC5539017

