# Supplementary material for: Can MRI differentiate between atypical cartilaginous tumors and high-grade chondrosarcoma? A systematic review
Source: Acta Orthop. 2020 May 20;91(4):471–8. doi: 10.1080/17453674.2020.1763717 (PMC8023913; doi:10.1080/17453674.2020.1763717)
Supplement: Supplemental Material [file IORT_A_1763717_SM8402.pdf]

Supplementary data

Table 1. Search strategy

	PubMed search July 12, 2018	Results
#1	(“Histology”[Mesh] OR Histology[tiab] OR Histological[tiab] OR histopathology*[tiab] OR “pathology”[MeSH Terms] OR pathology[tiab] OR pathological[tiab])	1,227,694
#2	(“Magnetic Resonance Imaging”[Mesh] OR imaging[tiab] OR diagnostic imaging [Subheading] OR mri[tiab] OR DWI[tiab] OR MR scan*[tiab])	1,749,265
#3	(“Chondrosarcoma”[Mesh]) OR Chondrosarcom*[tiab] OR (cartilag*[tiab] AND (tumor*[tiab] OR tumour*[tiab] OR sarcom*[tiab])))	14,424
#4	#1 AND #2 AND #3	824
	**Embase search July 12, 2018**	**Results**
#1	histology/ or pathology/ or histopathology/ or (histology or histological or histopathology or pathology or pathological).ti,ab,kw.	2,549,953
#2	nuclear magnetic resonance imaging/ or (imaging or mri or dwi or MR scan*).ti,ab,kw.	1,354,712
#3	Chondrosarcoma/ or Cartilage tumor/ or (chondrosarcom* or (cartilag* AND (tumor* OR tumour*OR sarcom*)).ti,ab,kw.	18,163
#4	#1 AND #2 AND #3	1,698

Table 2. Quality assessment

	Corresponding items on STROBE checklist [a]									
Study	Study design (4)	Setting (5)	Participants (6)	Variables (7)	Data sources/ measurement (8)	Bias (9)	Statistics (12)	Participants (13)	Descriptive data (14)	Outcome data (15)
Crim 2015	+	+	+	+	+	+	+	+	+	+
Douis 2014	+	+	+	+	+	+	+	+	+	+
Douis 2015	+	+	+	+	+	+	+	+	+	+
Douis 2018	+	+	+	+	+	+	+	+	+	+
Errani 2017	+	+	+	+	+	+	+	+	+	+
Fayad 2015	+	+	+	+	+	–	+	+	+	+
Kang 2016	+	+	+	+	+	+	+	+	+	+
Lisson 2018	–	-	+	+	+	–	+	+	+	+
Liu 2017	+	+	+	+	+	+	+	+	+	+
MacSweeney 2003	+	+	-	+	+	+	-	+	+	+
Müller 2016	+	+	+	+	+	–	+	+	+	+
Welzel 2018	+	+	+	+	+	+	+	+	+	+
Yoo 2009	+	+	+	+	+	+	+	+	+	+
Yoshimura 2013	+	+	+	+	+	–	–	+	+	+

[a] (Numbers) correspond with item numbers of the STROBE checklist.

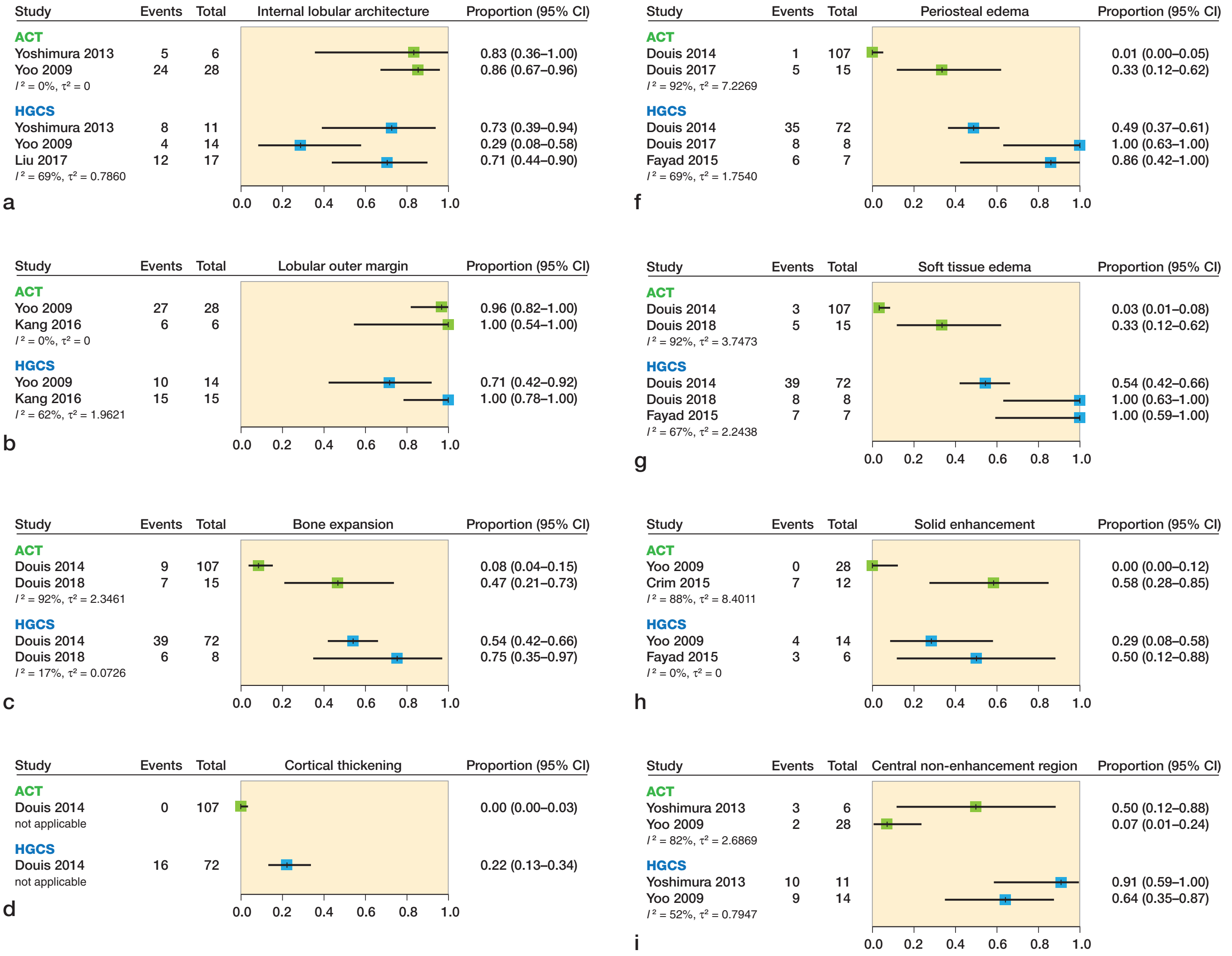

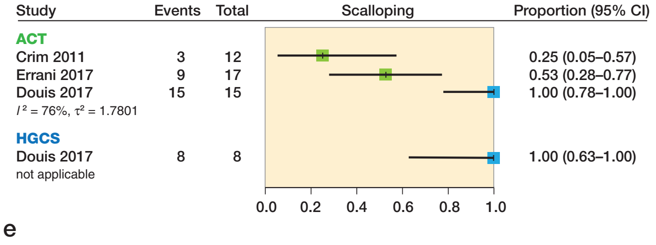

Figure 3. Forest plots of proportions of the reported presence of (a) internal lobular architecture, (b) lobular outer margin, (c) bone expansion, (d) cortical thickening, (e) scalloping, (f) periosteal edema, (g) soft tissue edema, (h) solid enhancement, and (i) central non-enhancing region on conventional MRI in atypical cartilaginous tumors (ACT) and high-grade chondrosarcoma (HGCS).

Table 4. Quality appraisal of pathological and MRI assessment

Study	A	B	C	D
Crim et al. 2015	Dahlin and Henderson 1956, Henderson and Dahlin 1963, Mirra et al. 1985, 1989, Brien et al. 1997	+ (blinded)	De Beuckeleer et al. 1995 and 1996, Geirnaerdt et al. 1997 and 2000, Murphey et al. 1998, 2003, Mirra et al. 1989, Walden et al. 2008	+ (blinded)
Douis et al. 2014	Evans et al. 1977, Dahlin and Beabout 1971, Enneking 1986	NR	–	+ (blinded to pathologic information)
Douis et al. 2015	Hogendoorn et al. 2013, Inwards 2013	+	–	+
Douis et al. 2018	Hoogendoorn et al. 2013	+	–	+
Errani et al. 2017	–	+	Murphey et al. 1998, Crim et al. 2015	– orthopedic oncologist
Fayad et al. 2015	Dorfman and Czerniak 1998	+ (imaging available)	–	+
Kang et al. 2016	Pritchard et al. 1980, Sanerkin 1980	NR	–	+ (blinded)
Lisson et al. 2018	–	NR	–	– experienced in MRI texture analysis
Liu et al. 2017	–	+	–	+
MacSweeney et al. 2003	Evans et al. 1977	NR	Mercuri and Campanacci 1995	*
Müller et al. 2016	–	NR	–	+ (blinded to pathologic and clinical information)
Welzel et al. 2018	Detailed description of the pathologic criteria is specified in the article	NR	–	+ (blinded to pathologic and clinical information)
Yoo et al. 2009	Welkerling et al. 1996, 2003, Rozeman et al. 2006, Inwards and Unni 1995	+	Welkerling et al. 2003, Geirnaerdt et al. 1993 and 2000, De Beuckeleer et al. 1995 and 1996, Murphey et al. 2003, Aoki et al. 1991	+ (blinded to pathologic information)
Yoshimura et al. 2013	Evans et al. 1977	NR	Murphey et al. 2003, Yoo et al. 2009	NR

A. Text includes reference to previously published or consensus criteria used for pathologic diagnosis.
B. Diagnosis established by a pathologist with expertise in musculoskeletal oncology.
C. Text includes reference to previously published or consensus criteria used for radiologic diagnosis.
D. Was the diagnosis established by a radiologist with expertise in musculoskeletal oncology.
NR = Not reported.
